# High burden and genetic diversity of β-lactamase-producing *Escherichia coli* and *Klebsiella pneumoniae* causing community-acquired urinary tract infections in Southeastern Gabon

**DOI:** 10.1371/journal.pone.0343632

**Published:** 2026-02-24

**Authors:** Yann Mouanga-Ndzime, Cyrille Bisseye, Annicet-Clotaire Dikoumba, Désiré Otsaghe Ekore, Michelle Bignoumba, Marie-Louise Mawili Mounguengui, Neil-Michel Longo-Pendy, Sylvain Godreuil, Barthélémy Ngoubangoye, Bolni Marius Nagalo, Richard Onanga

**Affiliations:** 1 Bacteriology Unit and One Health Laboratory, Interdisciplinary Centre for Medical Research of Franceville, Franceville, Gabon; 2 Research Unit in Biological Sciences (URSB), Laboratory of Molecular and Cellular Biology, University of Science and Technology of Masuku (USTM), Franceville, Gabon; 3 Omar Bongo Ondimba Army Instruction Hospital (HIAOBO), Libreville, Gabon; 4 Research Unit for the Ecology of Health, Interdisciplinary Centre for Medical Research of Franceville, Franceville, Gabon; 5 Laboratoire de Bactériologie, CHU de Montpellier, UMR MIVEGEC (IRD, CNRS, Université de Montpelier), Montpellier, France; 6 UMR MIVEGEC IRD-CNRS-Université de Montpellier, IRD, Montpellier, France; 7 Department of Pharmacology and Physiology, University of Maryland School of Medicine, Baltimore, Maryland, United States of America; 8 Marlene and Stewart Greenebaum NCI Comprehensive Cancer Center, University of Maryland School of Medicine, Baltimore, Maryland, United States of America; Rivers State University, NIGERIA

## Abstract

**Background:**

The increasing prevalence of β-lactamase-producing *Escherichia coli* and *Klebsiella pneumoniae* in community-acquired urinary tract infections (CA-UTIs) represents a growing public health challenge in low-resource settings. Data from Central Africa remain limited. This study investigated antimicrobial resistance profiles and phylogenetic diversity of these pathogens in southeastern Gabon.

**Methods:**

Patients presenting with CA-UTIs were screened, and isolates of *E. coli* and *K. pneumoniae* were collected. Antimicrobial susceptibility testing was performed using the Kirby–Bauer disk diffusion method and interpreted according to EUCAST 2024 guidelines. Phenotypic screening identified extended-spectrum β-lactamase (ESBL), AmpC, and carbapenemase production. Polymerase chain reaction (PCR) was used to detect ESBL genes (*bla-CTX-M-gp1, bla-CTX-M-gp2, bla-CTX-M-gp9, bla-TEM, bla-SHV*), plasmid-mediated AmpC genes (*bla-CMY-1, bla-CMY-2, bla-ACT-1, bla-ACC, bla-FOX, bla-DHA*), and the carbapenemase gene *bla-OXA-48*. Phylogenetic grouping of ESBL-producing *E. coli* was determined using PCR targeting *chuA, yjaA, Tsp*E4.C2, and *arpA.*

**Results:**

Among 3,026 screened patients, 949 CA-UTIs were confirmed, including 589 cystitis and 360 pyelonephritis cases, yielding 200 isolates: *E. coli* (124) and *K. pneumoniae* (76). Of these, 68 (34.0%) were ESBL producers, 17 (9.0%) AmpC producers, and 2 (1.0%) carbapenemase producers. ESBL producing isolates were significantly associated with male sex (47.0%, p = 0.02) and children ≤5 years of age (48.2%, p = 0.007). ESBL- and AmpC-producing isolates exhibited high resistance rates (20–100%) to β-lactams, fluoroquinolones, and trimethoprim–sulfamethoxazole, while retaining susceptibility to carbapenems, ceftazidime–avibactam, and nitrofurantoin (up to 100%). Molecular analysis showed predominance of *bla-*CTX-M-gp1 in *E. coli* (40% alone; 31% in combination with *bla-TEM*). In *K. pneumoniae, bla-SHV* *+ bla-TEM* (49%) and *bla-SHV* *+ bla-CTX-M-gp1* + *bla-TEM* (42%) were the most frequent gene combinations; one isolate carried *bla-OXA-48*. ESBL-producing *E. coli* predominantly belonged to phylogroup A (48.6%).

**Conclusion:**

Multidrug-resistant ESBL-, AmpC-, and OXA-48-producing *E. coli* and *K. pneumoniae* are circulating in community settings in southeastern Gabon. These findings underscore the need for strengthened antimicrobial resistance surveillance and evidence-based community-level interventions.

## Introduction

Community-acquired urinary tract infections (CA-UTIs) are among the most frequent bacterial infections worldwide, disproportionately affecting women, young children, and older adults. Management of CA-UTIs commonly relies on empirical antibiotic therapy initiated before microbiological results are available. This practice remains particularly widespread in sub-Saharan Africa, where limited laboratory infrastructure and shortages of trained personnel restrict access to routine bacteriological diagnostics. While empiric treatment is often clinically necessary, it exerts substantial selective pressure that facilitates the emergence and dissemination of multidrug-resistant (MDR) bacteria [1,2].

Among the most clinically significant resistance mechanisms in *Enterobacteriaceae* is the production of extended-spectrum β-lactamases (ESBLs). These enzymes hydrolyze penicillins, third-generation cephalosporins, and aztreonam, thereby conferring resistance to several first-line antibiotics commonly used for UTI treatment. ESBLs were initially described in *Klebsiella pneumoniae* in hospital settings and were predominantly associated with TEM- and SHV-type enzymes [3]. Since the early 2000s, however, a major epidemiological shift has occurred with the global emergence of community-acquired *Escherichia coli* producing CTX-M–type ESBLs, which now represent the most prevalent ESBL family associated with urinary tract infections worldwide [4,5].

Concurrently, the increasing detection of carbapenemase-producing *Enterobacteriaceae*, particularly those harboring OXA-48–type enzymes, represents a critical escalation in antimicrobial resistance. Although initially confined to healthcare-associated infections, these enzymes have increasingly been identified in community-acquired isolates, severely limiting therapeutic options [6]. In addition, AmpC-type cephalosporinases contribute to resistance against broad-spectrum β-lactams and pose diagnostic challenges due to inducible or low-level expression and the limited sensitivity of routine phenotypic assays [7].

In this context of expanding resistance mechanisms, the effectiveness of empirical therapy for CA-UTIs is becoming increasingly uncertain, especially in settings where local antimicrobial resistance data are sparse or unavailable. Comprehensive surveillance of resistance phenotypes, alongside molecular characterization of circulating β-lactamase genes, is essential for informing evidence-based treatment guidelines, minimizing therapeutic failure, and supporting antimicrobial stewardship initiatives at the community level.

In Gabon, and particularly in the southeastern region of the country, data on antimicrobial resistance among community-acquired *E. coli* and *K. pneumoniae* causing urinary tract infections remain limited. This study aimed to address this knowledge gap by determining the prevalence of β-lactamase–producing strains, characterizing their antimicrobial resistance profiles, and describing the distribution of major resistance genes in *E. coli* and *K. pneumoniae* isolated from community-acquired UTIs.

## Materials and methods

### Study design and study population

This cross-sectional study was conducted from January 2019 to December 2023 at the microbiology laboratory of the Interdisciplinary Centre for Medical Research of Franceville (CIRMF), located in Franceville, the capital city of Haut-Ogooué Province in southeastern Gabon. Franceville has an estimated population of approximately 250,000 inhabitants and shares a border with the Republic of Congo. The study population consisted of outpatients and patients hospitalized for ≤48 hours who underwent a cytobacteriological examination of urine (ECBU). Only community-acquired urinary tract infections (CA-UTIs) were included, defined as UTIs occurring in patients without recent hospitalization, urinary catheterization, or invasive urological procedures. Eligible participants included individuals of both sexes presenting a valid medical prescription from a licensed healthcare provider. Written informed consent was obtained from adult participants or from legal guardians for minors. Patients were stratified into four age groups: children ≤5 years, children 6–17 years, adults 18–49 years, and adults ≥50 years.

### Sample collection

Urine samples were preferentially collected in the morning or after a minimum of four hours without urination, either at the laboratory or at home, under strict aseptic conditions. Samples were collected in sterile, single-use containers. For children unable to use toilet facilities independently, clean-catch urine collection was preferred; when not feasible, sterile adhesive urine collection bags were used with assistance from parents, caregivers, or nursing staff. Following collection, samples were properly sealed, labeled with the time of collection, and transported by the patient to the laboratory at room temperature. Clinical information, including urinary symptoms and relevant medical history, was provided by the attending clinician on the laboratory request form. Socio-demographic data were collected using a structured questionnaire.

### Culture and identification of bacterial isolates

Urine culture and bacterial identification were performed as previously described [8]. Briefly, 10 µL of well-mixed urine was aseptically inoculated using a sterile disposable loop in a biosafety level 2 cabinet onto cystine–lactose–electrolyte-deficient (CLED) agar and MacConkey agar (bioMérieux, France). Samples were processed within two hours of collection to minimize contamination. Plates were incubated aerobically at 37 °C for 18–24 hours. According to Kass criteria, bacterial growth ≥10⁵ colony-forming units (CFU)/mL was considered significant, whereas counts <10⁵ CFU/mL or the presence of more than two bacterial species were considered indicative of contamination [9,10]. Colony counts were interpreted independently of patient sex or bacterial species. Preliminary identification was performed using Gram staining, oxidase, and catalase tests. Definitive identification to the genus and species level was achieved using conventional biochemical methods with the VITEK® 2 automated system (bioMérieux, Marcy-l’Étoile, France), following the manufacturer’s instructions [11]. Pure isolates of *E. coli* and *K. pneumoniae* were preserved in brain–heart infusion broth (Oxoid, UK) supplemented with 20% glycerol and stored at −80 °C for subsequent analyses.

### Antibiotic susceptibility testing

A total of 200 isolates (124 *E. coli* and 76 *K. pneumoniae*) were included in antimicrobial susceptibility testing. Frozen isolates were subcultured on blood agar and incubated at 37 °C for 24 hours. Susceptibility testing was performed using the Kirby–Bauer disk diffusion method on Mueller–Hinton agar and interpreted according to EUCAST 2024 guidelines [12]. Quality control was ensured using *E. coli* ATCC 25922 and *K. pneumoniae* ATCC 700603 reference strains [12]. The following antibiotics were tested: ampicillin (AMP, 10 µg), ticarcillin (TIC, 75 µg), amoxicillin–clavulanic acid (AMC, 20/10 µg), piperacillin–tazobactam (TZP, 30/6 µg), cephalothin (KF, 30 µg), cefoxitin (FOX, 30 µg), cefotaxime (CTX, 5 µg), ceftazidime (CAZ, 10 µg), ceftazidime–avibactam (CZA, 10/4 µg), aztreonam (ATM, 30 µg), ceftriaxone (CRO, 30 µg), cefepime (FEP, 30 µg), imipenem (IPM, 10 µg), gentamicin (GEN, 10 µg), tobramycin (TOB, 10 µg), ofloxacin (OFX, 5 µg), ciprofloxacin (CIP, 5 µg), nalidixic acid (NAL, 30 µg), trimethoprim–sulfamethoxazole (SXT, 1.25/23.75 µg), and nitrofurantoin (F, 100 µg).

### Phenotypic detection of β-lactamases

Extended-spectrum β-lactamase (ESBL) production was screened using ChromID® ESBL agar (bioMérieux) and confirmed by the double-disk synergy test (DDST). Disks containing cefotaxime, ceftazidime, and cefepime were placed 30 mm from an amoxicillin–clavulanic acid disk. ESBL production was confirmed by the appearance of a characteristic synergy (“champagne-cork” effect). In cases of suspected high-level cephalosporinase activity, a combined DDST using cloxacillin-supplemented media was performed [12]. AmpC β-lactamase production was detected using cephalosporin disks alone or in combination with cloxacillin on blood-supplemented Mueller–Hinton agar. Carbapenemase production was initially screened using ChromID® CARBA SMART agar (bioMérieux). Presumptive positive isolates were confirmed using an immunochromatographic assay (CORIS® RESIST-5 O.K.N.V.I.; Coris BioConcept), targeting KPC, OXA-48, NDM, VIM, and IMP enzymes.

### Detection of resistance genes and phylogenetic typing

Detection of β-lactamase genes *bla*-CTX-M (groups 1, 2, and 9), *bla*-TEM, *bla*-SHV, *bla*-FOX, and *bla*-DHA was performed as previously described [13]. Additional primers targeting *bla*-OXA-48, *bla*-ACC, *bla*-CMY-1, *bla*-CMY-2, and *bla*-ACT-1 were designed in-house using Primer3 and validated by BLAST analysis. Primer sequences and expected amplicon sizes are provided in S1 Table. Genomic DNA was extracted using the boiling method [14]. PCR amplification conditions are detailed in Supplementary Methods. Amplicons were visualized by electrophoresis on 1.5% agarose gels stained with GelRed® (Biotium), using a 100–1500 bp DNA ladder (Promega). Reference strains served as positive controls, and sterile water was used as a negative control. Phylogenetic grouping of ESBL-producing *E. coli* isolates was performed using the new Clermont quadruplex method. PCR-based method targeting *chu*A, *yja*A, *Tsp*E4.C2, and *arp*A, allowing classification into phylogroups A, B1, B2, C, D, E, F, or clade I [15]. PCR amplification conditions are detailed in Supplementary Methods.

### Ethical considerations

This study was conducted using archived, cryopreserved bacterial isolates previously collected during a UTI research project conducted between July 9, 2019, and October 8, 2023. Ethical approval for the original study was obtained from the Ethics Committee of the National Center for Scientific and Technological Research (CENAREST), Gabon (authorization no. AR0033/17/MESRSFC/CENAREST/CG/CST/CSAR, April 7, 2017). CENAREST oversees national research institutions, including CIRMF, where the study was performed. Data and samples were accessed for the present analysis in September 2024. All data were fully anonymized, and no identifiable patient information was accessible to the investigators. No additional human participants were recruited.

### Statistical analysis

Statistical analyses were performed using the R software. Categorical variables were compared using the Chi-square (χ²) test or Fisher’s exact test where appropriate. Logistic regression was used to assess the impact of the socio-clinical parameters of patients on the occurrence of cystitis and pyelonephritis in univariate and multivariate analyses. Odds ratios (ORs) and 95% confidence intervals are presented. A p-value of less than 0.05 was considered statistically significant.

## Results

### Screening, selection of urine samples, and identification of priority pathogens

**Fig 1:**
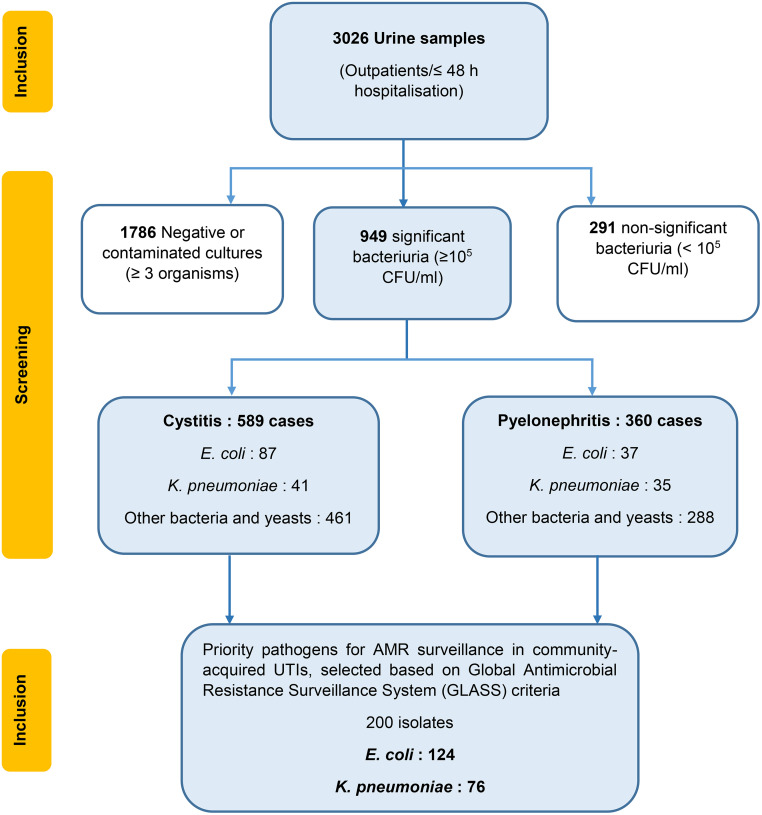
Flow chart of urine sample screening and selection of priority pathogens. A total of 3,026 urine samples were collected from outpatients or patients hospitalized for ≤48 hours. Of these, 1,786 samples yielded negative or contaminated cultures (≥3 organisms), and 291 showed non-significant bacteriuria (<10⁵ CFU/mL). Significant bacteriuria (≥10⁵ CFU/mL) was identified in 949 samples, which were clinically classified as cystitis (589 cases) or pyelonephritis (360 cases). Based on Global Antimicrobial Resistance Surveillance System (GLASS) criteria, 200 isolates were selected as priority pathogens for antimicrobial resistance surveillance in community-acquired urinary tract infections, comprising 124 *E. coli* and 76 *K. pneumoniae* isolates. CFU, colony-forming units; AMR, antimicrobial resistance; GLASS, Global Antimicrobial Resistance Surveillance System.

A total of 3,026 urine samples were collected from outpatients and patients hospitalized for ≤48 hours during the study period. Of these, 1,786 samples (59.0%) yielded negative or contaminated cultures, 291 (9.6%) showed non-significant bacteriuria (<10⁵ CFU/mL), and 949 (31.4%) demonstrated significant bacteriuria (≥10⁵ CFU/mL). Among the 949 confirmed urinary tract infections, 589 cases (62.0%) were clinically classified as cystitis and 360 cases (38.0%) as pyelonephritis. From the pool of samples with significant bacteriuria, *E. coli* and *K. pneumoniae* accounted for 200 isolates selected as priority uropathogens for antimicrobial resistance surveillance. The complete screening and selection process is summarized in Fig 1.

### General patient information in the study

Over the five-year study period, women accounted for 58.5% (1,769/3,026) of the study population, yielding a male-to-female ratio of 0.71 (Table 1). The overall mean age was 23.37 ± 19.89 years. Among pediatric participants, the median age was 2 years (interquartile range [IQR], 0.75–7), while adults had a median age of 33 years (IQR, 27–39). In the older adult group (≥50 years), the median age was 57 years (IQR, 53–65). Participants aged 18–49 years represented the largest age group, accounting for 45.0% (1,363/3,026) of all samples. Most participants resided in urban areas (71.4%), corresponding to an urban-to-rural ratio of 2.5 (Table 1). A total of 949 UTI cases (31.4%) were confirmed microbiologically. Among these, 589 cases (62.0%) were classified as cystitis and 360 cases (38.0%) as pyelonephritis. Negative or contaminated cultures represented 59.0% of all samples analyzed (Table 1). Among cultures with significant bacteriuria, *E. coli* was isolated in 124 cases (13.1%), with 70.0% of these isolates obtained from patients with cystitis. *K. pneumoniae* accounted for 76 isolates (8.0%), of which 54.0% were associated with cystitis and 46.0% with pyelonephritis (Table 1).

**Table 1:** Univariate and multivariate logistic regression analyses of cystitis and pyelonephritis according to sociodemographic, clinical, and seasonal factors.

Characteristics	UTIs										
	All patients (n = 3026)	Cystitis (n = 589)	cOR (95% CI)	*p-*value	aOR (95% CI)	*p-*value	Pyelonephritis (n = 360)	cOR (95% CI)	*p*-value	aOR (95% CI)	*p*-value
**Age groups (years)**											
[0-5]	944 (31%)	165 (17%)	0.50 (0.32-0.78)	0.003	0.48 (0.30-0.76)	0.002	174 (18%)	1.98 (1.27-3.10)	0.003	2.23 (1.13-4.41)	0.02
[6-17]	395 (13%)	70 (18%)	0.77 (0.44-1.31)	0.34	–	–	49 (12%)	1.29 (0.75-2.22)	0.340	–	–
[18–49]	1363 (45%)	283 (21%)	0.93 (0.69–1.25)	0.64	–	–	102 (7%)	0.66 (0.44–1.00)	0.05	–	–
≥ 50 (ref)	324 (11%)	71 (22%)	–	–	–	–	35 (11%)	–	–	–	
**Sex**											
Male (ref)	1257 (42%)	207 (16%)	–	–	–	–	149 (12%)	–	–	–	
Female	1769 (58%)	382 (22%)	1.39 (1.15–1.68)	<0.001	0.70 (0.58–0.85)	<0.001	211 (12%)	1.00 (0.80–1.25)	0.95	–	–
**Origin**											
Urban	2162 (71%)	440 (20%)	1.22 (0.99–1.50)	0.05	–	–	263 (12%)	1.09 (0.85–1.40)	0.47	–	–
Rural (ref)	864 (29%)	149 (17%)	–	–	–	–	97 (11%)	–	–	–	
**Season**											
Long dry	884 (29%)	190 (21%)	1.14 (0.87–1.49)	0.32	–	–	81 (9%)	0.55 (0.39–0.76)	<0.001	0.52 (0.37–0.73)	<0.001
Short dry (ref)	543 (18%)	105 (19%)	–	–	–	–	84 (15%)	–	–	–	
Long rainy	755 (25%)	132 (17%)	0.88 (0.66–1.17)	0.39	–	–	105 (14%)	0.88 (0.64–1.20)	0.43	–	–
Short rainy	844 (28%)	162 (19%)	0.99 (0.75–1.30)	0.94	–	–	90 (11%)	0.65 (0.47–0.89)	<0.01	0.59 (0.42–0.83)	<0.01
**Growth status**	3026 (100%)	–	–	–	–	–	–	–	–	–	–
Significant growth (≥1.0x10 ^**5**^ CFU/ml)	949 (31%)	589	–	–	–	–	360	–	–	–	–
*E. coli*	124 (4%)	87 (70%)	–	–	–	–	37 (30%)	–	–	–	–
*K. pneumoniae*	76 (2%)	41 (54%)	–	–	–	–	35 (46%)	–	–	–	–
Other bacteria and yeast	749 (25%)	461 (62%)					288 (38%)				–
Insignificant (<1.0x10 ^**5**^ CFU/ml)	291 (10%)	–	–	–	–	–	–	–	–	–	–
No growth and collection contamination (≥ 3 organisms)	1786 (59%)	–	–	–	–	–	–	–	–	–	–

*This table presents the results of univariate and multivariate logistic regression analyses evaluating the association between various parameters and the occurrence of cystitis and pyelonephritis. Crude and adjusted odds ratios (ORs) are reported for each variable. Crude ORs were calculated from the univariate analysis, while adjusted ORs were derived from the multivariate analysis, which controlled for the effects of all other variables included in the model. The associated p-values indicate the statistical significance of each association. The values in parentheses represent percentages, which indicate the proportion of patients in each category (e.g., age group, sex, origin) who had cystitis or pyelonephritis, relative to the total number of patients.*

### Patient characteristics stratified by β-lactamase production in community-acquired UTIs

A total of 200 community-acquired urinary tract infection cases caused by *E. coli* and *K. pneumoniae* were included in the analysis. Overall, 71.0% (142/200) of patients were female, corresponding to a male-to-female ratio of 0.40. The mean age was 21.66 ± 21.50 years. Among pediatric patients, the median age was 0.7 years (IQR, 0.43–2.0 years); the median age among adults was 34 years (IQR, 30–39), and among older adults was 55.5 years (IQR, 53–62). Children aged ≤5 years constituted the largest age group, accounting for 42.5% of cases. Most patients resided in urban areas (76.5%), and the majority reported no underlying comorbidities (94.5%, 189/200). Urinary symptoms were documented in 44.0% of patients (Table 2). Among the 200 isolates analyzed, 68 (34.0%) were identified as ESBL producers, including 35 *E. coli* (51.5%) and 33 *K. pneumoniae* (48.5%). ESBL production isolates were significantly more frequent among male patients (47.0%; p = 0.02) and among children aged ≤5 years (48.2%; p = 0.007). AmpC β-lactamase production was detected in 17 isolates (9.0%), comprising 3 *E. coli* (17.6%) and 14 *K. pneumoniae* (82.4%). AmpC-producing isolates were most commonly recovered from children aged ≤5 years (12.0%) and from pregnant women (25.0%). Carbapenem resistance was identified in two isolates (1.0%), both of which were *K. pneumoniae* (Table 2). Most resistant isolates originated from urban settings, accounting for 35.0% of ESBL producers, 10.4% of AmpC producers, and 1.3% of carbapenemase producers. With respect to comorbidities, 35.4% of ESBL-producing isolates were recovered from patients without reported underlying conditions. Clinically, urinary symptoms were frequently observed among patients infected with resistant organisms: 58.0% of ESBL-producing isolates (p < 0.0001) and 18.1% of AmpC-producing isolates were associated with symptomatic infections (Table 2).

**Table 2:** General characteristics of patients with community-acquired UTIs due to *E. coli* and *K. pneumoniae* isolates, stratified by β-lactamase production.

Characteristics	Total *E. coli* and *K. pneumoniae* isolates (n = 200)	ESBL-positive (n = 68)	*p-value*	AmpC-positive (n = 17)	*p-value*	Carbapenemase-positif (n = 2)	*p-value*
**Bacteria**							
*E. coli*	124 (62.0%)	35 (28.2%)	**0.04**	3 (2.4%)	**<0.001**	0 (0%)	–
*K. pneumoniae*	76 (38.0)	33 (43.4%)		14 (18.4%)		2 (2.6%)	
**Sex**							
Male	58 (29.0%)	27 (47.0%)	**0.02**	**7** (12.0%)	NS	1 (2.0%)	–
Female	142 (71.0%)	41 (29.0%)		10 (7.0%)		1 (0.7%)	
**Age groups**							
≤ 5	85 (42.5%)	41 (48.2%)	**<0.01**	10 (12.0%)	NS	1 (1.1%)	–
6-17	13 (6.5%)	2 (15.4%)		1 (8.0%)		0 (0.0%)	
18-49	76 (38.0%)	17 (22.3%)		5 (7.0%)		1 (1.3%)	
≥ 50	26 (13.0%)	8 (31.0%)		1 (4.0%)		0 (0.0%)	
**Origin**							
Urban area	153 (76.5%)	53 (35.0%)	NS	16 (10.4%)	NS	2 (1.3%)	–
Rural area	47 (23.5%)	15 (32.0%)		1 (2.1%)		0 (0.0%)	
**Underlying terrain**							
None	189 (94.5%)	67 (35.4%)	NS	15 (8.0%)	NS	2 (1.05%)	–
Comorbidity	7 (3.5%)	1 (14.2%)		1 (14.2%)		0 (0.0%)	
Pregnancy	4 (2.0%)	0 (0.0%)		1 (25.0%)		0 (0.0%)	
**Symptoms**							
Urinary signs	88 (44.0%)	51 (58.0%)	**<0.0001**	16 (18.1%)	NS	1 (1.1%)	–
Non-urinary signs	13 (6.5%)	1 (8.0%)		1 (8.0%)		1 (8.0%)	
None	99 (49.5%)	16 (16.1%)		0 (0.0%)		0 (0.0%)	

*This table presents the distribution of key demographic and clinical variables among all isolates (n = 200), as well as among subsets producing ESBL (n = 68), AmpC (n = 17), Carba (n = 2). Statistical comparisons between subgroups (e.g., male vs. female) within each β-lactamase type were performed using Chi-square or Fisher’s exact test. P-values are reported in the Results section. NS: not significant; –: not applicable. ESBL: extended-spectrum β-lactamase; AmpC: AmpC β-lactamase; Carba: carbapenemase.*

### Distribution of ESBL-producing *E. coli* and *K. pneumoniae* strains

Antibiotic resistance rates were significantly higher among ESBL-producing *E. coli* isolates compared with non-ESBL-producing isolates across multiple antibiotic classes (Table 3). ESBL-producing *E. coli* showed universal resistance to ampicillin (100% vs 62%; p < 0.001) and ticarcillin (100% vs 57%; p < 0.001). Resistance was also significantly higher to amoxicillin–clavulanic acid (69% vs 39%; p < 0.001), cephalothin (88% vs 23%; p < 0.001), cefotaxime (83% vs 14%; p < 0.001), ceftazidime (77% vs 10%; p < 0.001), and cefepime (63% vs 5%; p < 0.001). In addition to β-lactams, ESBL-producing *E. coli* isolates demonstrated significantly increased resistance to non–β-lactam agents, including nalidixic acid (83% vs 41%; p < 0.001), ciprofloxacin (63% vs 20%; p < 0.001), ofloxacin (80% vs 32%; p < 0.001), tobramycin (60% vs 17%; p < 0.001), and trimethoprim–sulfamethoxazole (80% vs 54%; p = 0.01). A similar resistance pattern was observed among *K. pneumoniae* isolates. ESBL-producing strains exhibited significantly higher resistance than non-ESBL-producing strains to amoxicillin–clavulanic acid (73% vs 42%; p = 0.02), cephalothin (97% vs 49%; p < 0.001), cefotaxime (97% vs 30%; p < 0.001), ceftazidime (70% vs 23%; p < 0.001), and cefepime (60% vs 16%; p < 0.001). Elevated resistance was also observed for ofloxacin (54% vs 23%; p = 0.01), gentamicin (76% vs 11%; p < 0.001), and tobramycin (67% vs 7%; p < 0.001) (Table 3).

**Table 3:** Comparison of resistance rates to commonly used antibiotics between ESBL-producing and non-ESBL-producing *Escherichia coli* and *Klebsiella pneumoniae* isolates from community-acquired urinary tract infections.

Antibiotics	*E. coli* (N = 124)		*p* -value	*K. pneumoniae* (N = 76)		*p* -value
	ESBL			ESBL		
	ESBL (n = 35)	Non-ESBL (n = 89)		ESBL (n = 33)	Non-ESBL (n = 43)	
**β-lactams**						
Ampicillin	35 (100%)	55 (62%)	**<0.001**	33 (100%)	43 (100%)	–
Ticarcillin	35 (100%)	51 (57%)	**<0.001**	33 (100%)	43 (100%)	–
Amoxicillin- clavulanic acid	24 (69%)	35 (39%)	**<0.001**	24 (73%)	18 (42%)	**0.02**
Piperacilline-tazobactam	7 (20%)	16 (19%)	NS	10 (30%)	6 (14%)	NS
Cefalotin	31 (88%)	21 (23%)	**<0.001**	32 (97%)	21 (49%)	**<0.001**
Cefoxitin	3 (8%)	9 (10%)	NS	6 (18%)	16 (37%)	NS
Cefotaxime	29 (83%)	13 (14%)	**<0.001**	32 (97%)	13 (30%)	**<0.001**
Ceftazidime	27 (77%)	9 (10%)	**<0.001**	23 (70%)	10 (23%)	**<0.001**
Ceftazidime-avibactam	0 (0%)	0 (0%)	**–**	0 (0%)	0 (0%)	**–**
Cefepime	22 (63%)	5 (5%)	**<0.001**	20 (60%)	7 (16%)	**<0.001**
Imipenem	1 (3%)	0 (0%)	NS	1 (3%)	2 (5%)	NS
Ertapenem	1 (3%)	1 (1%)	NS	1 (3%)	4 (9%)	NS
**Quinolones** **and fluoroquinolones**						
Nalidixic acid	29 (83%)	37 (41%)	**<0.001**	10 (30%)	12 (28%)	NS
Ciprofloxacin	22 (63%)	18 (20%)	**<0.001**	7 (21%)	3 (7%)	NS
Ofloxacin	28 (80%)	29 (32%)	**<0.001**	18 (54%)	10 (23%)	**0.01**
**Aminoglycosides**						
Gentamicin	14 (40%)	43 (48%)	NS	25 (76%)	5 (11%)	**<0.001**
Tobramycin	21 (60%)	15 (17%)	**<0.001**	22 (67%)	3 (7%)	**<0.001**
Amikacin	3 (8%)	1 (1%)	NS	2 (6%)	0 (0%)	NS
**Sulfonamides**						
Trimethoprim-Sulfamethoxazole	28 (80%)	48 (54%)	**0.01**	21 (64%)	31 (72%)	NS
**Nitrofurans**						
Nitrofurantoin	0 (0%)	1 (1%)	NS	0 (0%)	1 (2%)	NS

*Resistance percentages are shown for each group. Statistical comparisons were performed using Chi-square or Fisher’s exact test. P-values are reported in the table.*

*ESBL: extended-spectrum β-lactamase; NS: not significant; –: not applicable (all values equal in both groups).*

### Distribution of AmpC-producing *E. coli* and *K. pneumoniae*

Phenotypic antimicrobial resistance among AmpC-producing *E. coli* isolates was evaluated based on three strains (Table 4). Compared with non-AmpC-producing *E. coli*, AmpC-producing isolates exhibited significantly higher resistance to cefoxitin (100% vs 7%; p < 0.001), ceftazidime (100% vs 27%; p = 0.03), imipenem (33% vs 0%; p = 0.001), ertapenem (33% vs 0.8%; p = 0.03), and nitrofurantoin (33% vs 0%; p = 0.001). Similarly, AmpC-producing *K. pneumoniae* isolates demonstrated significantly higher resistance rates than non-AmpC-producing isolates to several antibiotics, including amoxicillin–clavulanic acid (100% vs 45%; p < 0.001), cephalothin (100% vs 63%; p = 0.01), cefoxitin (100% vs 13%; p < 0.001), ceftazidime (71% vs 37%; p = 0.04), and nalidixic acid (64% vs 21%; p < 0.01) (Table 4).

**Table 4:** Comparison of resistance rates to commonly used antibiotics between AmpC-producing and non-AmpC-producing *Escherichia coli* and *Klebsiella pneumoniae* isolates from community-acquired urinary tract infections.

Antibiotics	*E. coli* (n = 124)		*p* -value	*K. pneumoniae* (n = 76)		*p* -value
	AmpC			AmpC		
	AmpC (n = 3)	Non-AmpC (n = 121)		AmpC (n = 14)	Non-AmpC (n = 62)	
**β-lactams**						
Ampicillin	3 (100%)	87 (72%)	NS	14 (100%)	62 (100%)	–
Ticarcillin	3 (100%)	83 (68%)	NS	14 (100%)	62 (100%)	–
Amoxicillin-clavulanic acid	3 (100%)	56 (46%)	NS	14 (100%)	28 (45%)	**<0.001**
Piperacillin-tazobactam	1 (33%)	22 (18%)	NS	3 (21%)	13 (21%)	NS
Cefalotin	3 (100%)	49 (40%)	NS	14 (100%)	39 (63%)	**0.01**
Cefoxitin	3 (100%)	9 (7%)	**<0.001**	14 (100%)	8 (13%)	**<0.001**
Cefotaxime	3 (100%)	39 (32%)	NS	10 (71%)	35 (56%)	NS
Ceftazidime	3 (100%)	33 (27%)	**0.03**	10 (71%)	23 (37%)	**0.04**
Ceftazidime-avibactam	0 (0%)	0 (0%)	**–**	0 (0%)	0 (0%)	**–**
Cefepime	2 (66%)	25 (20%)	NS	3 (21%)	24 (39%)	NS
Imipenem	1 (33%)	0 (0%)	**0.001**	2 (14%)	1 (1%)	NS
Ertapenem	1 (33%)	1 (1%)	**0.03**	1 (7%)	4 (6%)	NS
**Quinolones** **and fluoroquinolones**						
Nalidixic acide	2 (66%)	64 (53%)	NS	9 (64%)	13 (21%)	**<0.01**
Ciprofloxacin	2 (66%)	38 (31%)	NS	1 (7%)	9 (14%)	NS
Ofloxacin	3 (100%)	54 (44%)	NS	9 (64%)	19 (30%)	NS
**Aminoglycosides**						
Gentamicin	2 (66%)	55 (45%)	NS	6 (43%)	24 (39%)	NS
Tobramycin	2 (66%)	34 (28%)	NS	4 (28%)	21 (34%)	NS
Amikacin	1 (33%)	3 (2%)	NS	0 (0%)	2 (3%)	NS
**Sulfonamides**						
Trimethoprim-Sulfamethoxazole	2 (66%)	74 (61%)	NS	13 (93%)	39 (63%)	NS
**Nitrofurans**						
Nitrofurantoin	1 (33%)	0 (0%)	**0.001**	1 (7%)	0 (0%)	NS

*Resistance percentages are shown for each group. Statistical comparisons were performed using Chi-square or Fisher’s exact test. P-values are reported in the table.*

*AmpC: AmpC β-lactamase; NS: not significant; –: not applicable (all values equal in both groups).*

### Prevalence of β-lactamase-encoding genes in *E. coli* and *K. pneumoniae*

**Fig 2:**
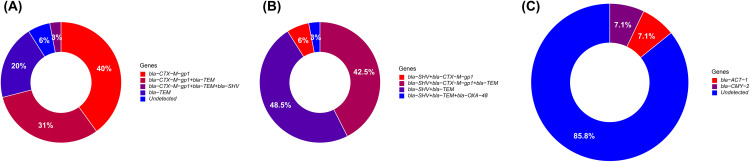
Distribution of β-lactamase-encoding genes in *E. coli* and *K. pneumoniae.* This figure illustrates the distribution of beta-lactamase gene combinations identified in *E. coli* and *K. pneumoniae* isolates. A total of 35 *E. coli* and 33 *K. pneumoniae* isolates producing Extended-Spectrum β-lactamase (ESBL) were analyzed. In *E. coli*, the most frequent gene combinations included bla-CTX-M-gp1 alone or associated with bla-TEM, whereas in *K. pneumoniae*, profiles combining bla-SHV, bla-CTX-M-gp1, and bla-TEM were predominant. Carbapenemase genes were investigated in *K. pneumoniae* and the bla-OXA-48 gene was detected in one isolate. AmpC β-lactamase genes were assessed in 14 *K. pneumoniae* isolates, in which bla-CMY-2 and bla-ACT-1 were identified, while no AmpC genes were detected in *E. coli*. Some isolates showed no β-lactamase gene detected by the panel used.

PCR-based screening for major β-lactamase–encoding genes revealed heterogeneous genetic profiles among ESBL-producing isolates. The genes *bla*-CTX-M-gp1, *bla-*TEM, and *bla*-SHV were detected either alone or in combination, whereas *bla*-CTX-M-gp2 and *bla*-CTX-M-gp9 were not identified in any isolate. Among ESBL-producing *E. coli* isolates (n = 35), 40.0% (14/35) carried *bla-*CTX-M-gp1 alone, while 20.0% (7/35) harbored *bla*-TEM alone. Co-carriage of *bla*-CTX-M-gp1 and *bla*-TEM was observed in 31.0% (11/35) of isolates, and a triple gene combination (*bla*-CTX-M-gp1 + *bla*-TEM + *bla*-SHV) was detected in one isolate (3.0%). Two isolates (6.0%) did not carry any of the targeted ESBL genes (Fig 2). Among ESBL-producing *K. pneumoniae* isolates (n = 33), the most frequent genotype was the co-carriage of *bla*-SHV and *bla*-TEM, identified in 49.0% (16/33) of isolates. Two isolates (6.0%) carried *bla*-SHV + *bla*-CTX-M-gp1, while 42.0% (14/33) harbored a triple combination of *bla-*SHV + *bla*-CTX-M-gp1 + *bla*-TEM (Fig 2). Analysis of plasmid-mediated AmpC genes in *K. pneumoniae* revealed the presence of *bla*-CMY-2 in one isolate (7.1%, 1/14) and *bla*-ACT-1 in one isolate (7.1%, 1/14). No AmpC genes were detected in *E. coli*, and *bla*-CMY-1, *bla-*ACC, *bla*-FOX, and *bla-*DHA were not identified in either species. Finally, one *K. pneumoniae* isolate (3.0%, 1/33) carried a carbapenemase gene, *bla*-OXA-48, in combination with *bla*-SHV and *bla*-TEM (Fig 2).

### Distribution of phylogenetic groups among ESBL-producing *E. coli* strains

**Fig 3:**
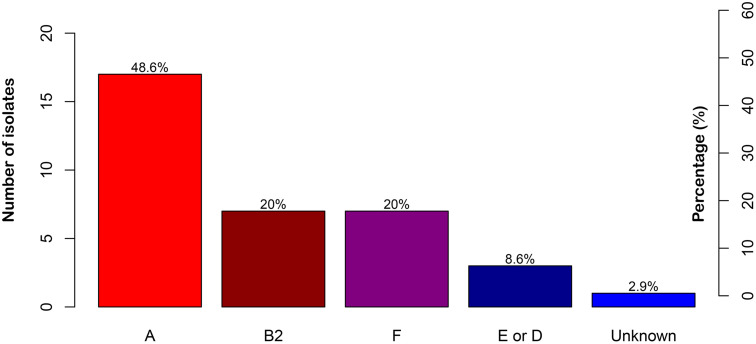
Distribution of *E. coli* phylogenetic groups. This figure shows the distribution of phylogenetic groups among 35 ESBL-positive *E. coli* isolates. Group A was the most prevalent, followed by groups B2 and **F.** A smaller proportion of isolates belonged to group E or D.

Among the 35 ESBL-producing *E. coli* isolates analyzed, phylogenetic group A was the most prevalent, accounting for 48.6% of cases (n = 17). Groups B2 and F were each identified in 20.0% of the isolates (n = 7). Three strains (8.6%) exhibited a genetic profile compatible with both groups E and D. Finally, one isolate (2.8%) could not be assigned to any known phylogenetic group (Fig 3).

## Discussion

The circulation of multidrug-resistant *E. coli* and *K. pneumoniae* in community-acquired urinary tract infections (CA-UTIs) represents a growing challenge for empirical therapy and antimicrobial resistance (AMR) surveillance. In this study conducted in southeastern Gabon, we focused on these two GLASS priority pathogens to characterize the prevalence, resistance phenotypes, and genetic determinants of ESBL-, AmpC-, and carbapenemase-producing strains circulating in the community. We observed that 34% of *E. coli* and *K. pneumoniae* isolates were ESBL producers. This prevalence is comparable to earlier hospital-based studies from Gabon reporting a predominance of ESBL-producing *Enterobacterales* in UTIs [16,17], but our findings extend these observations by demonstrating substantial circulation of these resistant strains outside healthcare settings. The presence of ESBL-producing organisms in community-acquired infections is of particular concern, as it complicates empirical treatment strategies and increases the risk of therapeutic failure, especially in settings with limited access to routine microbiological testing.

The proportion of ESBL-producing *E. coli* isolates (28%) was lower than those reported in Gabon [16], Egypt [18], Togo [19], and Ethiopia [20], but higher than rates observed in Cameroon, Tanzania, Benin, Central Europe, and the United States [21–25]. For *K. pneumoniae*, the ESBL prevalence (43%) was comparable to Mali [26], lower than Burkina Faso and Ethiopia [20,27], but higher than reports from Gabon, Cameroon, and Tanzania [16,21,22]. This heterogeneity likely reflects differences in antibiotic prescribing practices, β-lactam selection pressure in human and veterinary medicine, and variability in surveillance systems and infection control measures across regions. AmpC β-lactamase production was detected in 9% of isolates, a prevalence comparable to reports from Gabon, Egypt, and China [16,18,28], but higher than those observed in Europe and Oceania [29–31]. Among *E. coli*, the low prevalence of AmpC producers (2%) aligns with data from several countries [16,32,33], whereas the relatively high proportion observed in *K. pneumoniae* (18%) exceeds previous reports from Gabon and Europe [16,34,35]. Although AmpC producers remain a minority, their presence in community-acquired infections warrants attention due to their ability to compromise third-generation cephalosporins and β-lactam/β-lactamase inhibitor combinations.

Consistent with these findings, ESBL- and AmpC-producing isolates exhibited high resistance rates to first-line antibiotics, including β-lactams, fluoroquinolones, aminoglycosides, and trimethoprim–sulfamethoxazole. Resistance levels exceeded pooled estimates from Central Africa [36], indicating a particularly high local burden of multidrug resistance. In contrast, carbapenems, ceftazidime–avibactam, and nitrofurantoin retained high activity, likely reflecting restricted community access and predominantly parenteral use. The documented use of “Watch”-category antibiotics, such as gentamicin and fluoroquinolones, in southeastern Gabon may contribute to the observed resistance patterns [37]. Together, these findings emphasize the need for strengthened antimicrobial stewardship, routine susceptibility testing, and locally adapted empirical treatment guidelines.

At the molecular level, *bla*-CTX-M-gp1 was the predominant ESBL gene among *E. coli*, consistent with numerous studies across sub-Saharan Africa highlighting the widespread dissemination of CTX-M-type enzymes [16,27,38–40]. Its detection in community isolates underscores the importance of extending molecular surveillance beyond hospital settings. In *K. pneumoniae*, co-carriage of *bla*-SHV and *bla*-TEM was most frequent, often in combination with *bla-*CTX-M-gp1, reflecting accumulation of multiple resistance determinants. Similar gene combinations have been reported in the region and support the role of *K. pneumoniae* as a key reservoir for ESBL evolution and dissemination [40–42]. Among AmpC genes, *bla*-CMY-2 and *bla*-ACT-1 were each detected in a small proportion of *K. pneumoniae* isolates. While *bla*-CMY-2 is widely distributed and commonly associated with community infections [7], *bla*-ACT-1 is less frequently reported outside hospital settings [43]. The absence of *bla*-CMY-1, *bla*-ACC, *bla*-FOX, and *bla*-DHA contrasts with reports from Asia and Europe [44], suggesting a more restricted AmpC gene pool in this setting, though continued monitoring remains warranted. The detection of a community-acquired *K. pneumoniae* isolate harboring *bla*-OXA-48 is of particular concern. OXA-48 carbapenemases, first described in Turkey [45,46], have disseminated widely in North Africa and increasingly in sub-Saharan Africa, including Gabon [47–50]. Its identification in a community isolate suggests potential spillover of carbapenemase-producing organisms beyond hospital environments and highlights the need for early detection and containment strategies. Phylogenetic analysis revealed that nearly half of ESBL-producing *E. coli* belonged to phylogroup A, followed by B2 and F. This distribution differs from reports where phylogroup B2 predominates among UTI-associated *E. coli* [40,51,52], and may reflect local dynamics in which commensal phylogroup A strains acquire resistance plasmids. Similar patterns involving CTX-M-producing *E. coli* from phylogroups A and D have been described in African extra-intestinal infections [53–56]. The presence of B2 and F phylogroups suggests the possible circulation of more virulent lineages, potentially including ST131, although genomic analyses are required to confirm this.

Several limitations should be acknowledged. These include the small number of AmpC- and carbapenemase-producing isolates, reliance on archived strains, absence of detailed molecular typing (e.g., MLST or plasmid analysis), and lack of clinical outcome data. Despite these constraints, the study provides a comprehensive overview of community-associated multidrug-resistant *E. coli* and *K. pneumoniae* in southeastern Gabon and situates local findings within regional and global AMR trends.

## Conclusion

Multidrug-resistant *E. coli a*nd *K. pneumoniae,* including ESBL-, AmpC-, and OXA-48–producing strains, are now established in community-acquired urinary tract infections in southeastern Gabon. ESBL- and AmpC-producing isolates exhibited high resistance to β-lactams, fluoroquinolones, and trimethoprim–sulfamethoxazole, while carbapenems, ceftazidime–avibactam, and nitrofurantoin largely retained activity. The predominance of phylogroup A among ESBL-producing *E. coli*, alongside the presence of B2 and F lineages, suggests the coexistence of resistant commensal strains and potentially more virulent clones in the community. These findings underscore the need for strengthened community-level AMR surveillance, improved antimicrobial stewardship, and updated empirical treatment guidelines to limit further dissemination of high-risk *Enterobacterales* in southeastern Gabon.

## Supporting information

S1 File Supplementary data. (DOCX)

S2 File S1 raw image. (PDF)
