# Supplementary material for: High burden and genetic diversity of β-lactamase-producing Escherichia coli and Klebsiella pneumoniae causing community-acquired urinary tract infections in Southeastern Gabon
Source: PLoS One. 2026 Feb 24;21(2):e0343632. doi: 10.1371/journal.pone.0343632 (PMC12931754; doi:10.1371/journal.pone.0343632)
Supplement: S1 File — (DOCX) [file pone.0343632.s001.docx]

**Table S1:** Characteristics of simplex PCR assays for the detection of β-lactamase genes.

| **_PCR_** | **_β-Lactamase target_** | **_(5'-3') sequences_** | **_Primer concentration_**  **_(pmol/µL)_** | **_Amplicon size (bp)_** |
| --- | --- | --- | --- | --- |
| _Simplex PCR for ESBL genes_ | _TEM variants including TEM-1 and TEM-2_ | _CATTTCCGTGTCGCCCTTATTC_  _CGTTCATCCATAGTTGCCTGAC_ | _0.4_  _0.4_ | _800_ |
|  | _SHV variants including SHV-1_ | _AGCCGCTTGAGCAAATTAAAC_  _ATCCCGCAGATAAATCACCAC_ | _0.4_  _0.4_ | _713_ |
|  | _CTX-M group 1 variants including CTX-M-1, CTX-M-3 and CTX-M-15_ | _TTAGGAARTGTGCCGCTGYA*_  _CGATATCGTTGGTGGTRCCAT*_ | _0.4_  _0.2_ | _688_ |
|  | _CTX-M group 2 variants including CTX-M-2_ | _CGTTAACGGCACGATGAC_  _CGATATCGTTGGTGGTRCCAT*_ | _0.2_  _0.2_ | _404_ |
|  | _CTX-M group 9 variants including CTX-M-9 and CTX-M-14_ | _TCAAGCCTGCCGATCTGGT_  _TGATTCTCGCCGCTGAAG_ | _0.4_  _0.4_ | _561_ |
| _Simplex PCR for AmpC genes_ | _FOX-1 to FOX-5_ | _CTACAGTGCGGGTGGTTT_  _CTATTTGCGGCCAGGTGA_ | _0.5_  _0.5_ | _162_ |
|  | _DHA-1 to DHA-2_ | _TGATGGCACAGCAGGATATTC_  _GCTTTGACTCTTTCGGTATTCG_ | _0.5_  _0.5_ | _997_ |
|  | _ACC_ | _TACCTGTCTGGCAGC AACTG_  _TGTTGTAGCCCCACG CATAG_ | _0.2_ | _647_ |
|  | _CMY-1_ | _CGTGCTCAAGGATG GCAAG_  _GGTTGGCCAGCAT GACGA_ | _0.2_ | _920_ |
|  | _CMY-2_ | _TGATGCAGGAGCA GGCWA_  _ACAGACCAATGCTGGA GTTAG_ | _0.2_ | _417_ |
|  | _ACT-I_ | _GCATTACGCCTGGGGATAC_  _GACCGGCCAGTTAAGCAT_ | _0.2_ | _244_ |
| _Simplex PCR for Carba genes_ | _OXA-48_ | _CTTAAACGGGCGAAC CAAGC_  _CATACGTGCCTCR CCAAT_ | _0.2_ | _232_ |

Table S 2: Primer sequences and sizes of PCR products used in the phylo-typing method.

| **PCR reaction** | **Primer ID** | **Target** | **Primer sequences (5'-> 3')** | **bp** |
| --- | --- | --- | --- | --- |
| Quadruplex | chuA. 1b | *chu*A | ATGGTACCGGACGAACCAAC | 288 |
|  | chuA.2 |  | TGCCGCCAGTACCAAAGACA |  |
|  | yjaA. 1b | *yja*A | CAAACGTGAAGTGTCAGGAG | 211 |
|  | yjaA. 2b |  | AATGCGTTCCTCAACCTGTG |  |
|  | TspE4C2.1b | TspE4C2 | CACTATTCGTAAGGTCATCC | 152 |
|  | TspE4C2.2b |  | AGTTTATCGCTGCGGGTCGC |  |
|  | AceK.f | *arp*A | AACGCTATTCGCCAGCTTGC | 400 |
|  | ArpA1.r |  | TCTCCCCATACCGTACGCTA |  |
| Groupe E | ArpAgpE.f | *arp*A | GATTCCATCTTGTCAAAATATGCC | 301 |
|  | ArpAgpE.r |  | GAAAAGAAAAAGAATTCCCAAGAG |  |
| Groupe C | trpAgpC.1 | *trp*A | AGTTTTATGCCCAGTGCGAG | 219 |
|  | trpAgpC.2 |  | TCTGCGCCGGTCACGCCC |  |

***Legend:***

*The table summarizes the molecular characteristics of the primers employed in the extended quadruplex PCR and supplementary reactions for the identification of E. coli phylogroups. For each reaction, the primer identification (ID), the corresponding genetic target, the specific nucleotide sequences (5' →3'), and the expected amplicon sizes in base pairs (bp) are provided. The Quadruplex reaction targets the chuA, yjaA, and TspE4C2 DNA fragments, along with the arpA gene. The amounts of primer used are 20 pmol, except for AceK.f (40 pmol), ArpA1.r (40 pmol).*

**Supplementary Methods**

For the resistance genes *bla*-CTX-M-1, *bla*-CTX-M-2, *bla*-CTX-M-9, *bla*-TEM, *bla*-SHV, *bla*-FOX, and *bla*-DHA. PCR reactions were carried out in a final volume of 25 µL, containing 1 µL of template DNA, 14 µL of EmeraldAmp® MAX PCR Master Mix (Takara Bio Inc.), 1 µL of each primer (final concentration 0.2–0.5 µM), and ultrapure water. The thermal cycling conditions were: initial denaturation at 94 °C for 10 minutes; 30 cycles of denaturation at 94 °C for 40 seconds, annealing at 55–60 °C (depending on the primer pair) for 40 seconds, and extension at 72 °C for 1 minute; followed by a final extension step at 72 °C for 7 minutes.

For the resistance genes *bla*-OXA-48, *bla*-ACC, *bla*-CMY-1, *bla*-CMY-2, and *bla*-ACT-1, PCR reactions were prepared in a final volume of 50 µL, containing 2 µL of template DNA, 25 µL of 2x EmeraldAmp® MAX PCR Master Mix (Takara Bio Inc.), 1 µL of each primer (final concentration 0.2 µM), and 23 µL of ultrapure water. Amplification of *bla*-OXA-48 was performed with an initial denaturation at 94 °C for 5 minutes, followed by 30 cycles of denaturation at 94 °C for 25 seconds, annealing at 52 °C for 40 seconds, and extension at 72 °C for 50 seconds, with a final extension at 72 °C for 6 minutes. AmpC genes (*bla*-ACC, *bla*-CMY-1, *bla*-CMY-2, and *bla*-ACT-1) were amplified using an initial denaturation at 94 °C for 10 minutes, followed by 30 cycles of denaturation at 94 °C for 40 seconds, annealing at 60 °C for 40 seconds, and extension at 72 °C for 60 seconds, with a final extension at 72 °C for 7 minutes.

For Phylogenetic grouping of *E. coli*, the PCR Cycling conditions included an initial denaturation at 94 °C for 5 minutes, followed by 30 cycles of denaturation at 94 °C for 30 seconds, annealing at 59 °C for 30 seconds, and extension at 72 °C for 30 seconds, with a final elongation at 72 °C for 7 minutes.

**S1 raw image.**

**Title**

Representative agarose gel electrophoresis of PCR amplicons for resistance genes and phylogenetic typing.

**Legend**

The figure illustrates the visual confirmation of PCR amplification for the targeted $\beta$-lactamase genes and the Clermont phylogenetic groups. In all panels, **M** represents the molecular weight marker (100 bp DNA ladder), **T-** corresponds to the negative control, and **T+** corresponds to the positive control. **(A)** PCR amplification of the ***bla*-CTX-M-group 1** gene showing the expected bands at **688 bp**. **(B)** Amplification of the ***bla*-TEM** gene with bands at the expected size of **800 bp**. **(C)** Amplification of the ***bla*-SHV** gene with bands at the expected size of **713 bp**. **(D)** Amplification of the ***bla*-ACT-1** gene (AmpC) showing the characteristic band at **244 bp**. **(E)** Specific amplification of the ***bla*-OXA-48** carbapenemase gene with the expected product at **232 bp**. **(F)** Amplification of the ***bla*-CMY-2** gene at the expected size of **417 bp**. **(G)** Quadruplex PCR profiles using the new Clermont phylo-typing method; lanes 1-5, 7,10-11 (*arpA+*) and 8-9 (*yjA*+ and *arpA+*) demonstrate profiles for **phylogroup A**. **(H)** Quadruplex PCR profiles for phylogenetic identification; lanes 1, 3, 4, and 5 represent **group A** (*arpA*+), while lanes 2, 6, 8, and 9 represent **group B2** (*chuA+* and *yjaA*+).
