# Supplementary figures and images for: High burden and genetic diversity of β-lactamase-producing Escherichia coli and Klebsiella pneumoniae causing community-acquired urinary tract infections in Southeastern Gabon
Source: PLoS One. 2026 Feb 24;21(2):e0343632. doi: 10.1371/journal.pone.0343632 (PMC12931754; doi:10.1371/journal.pone.0343632)

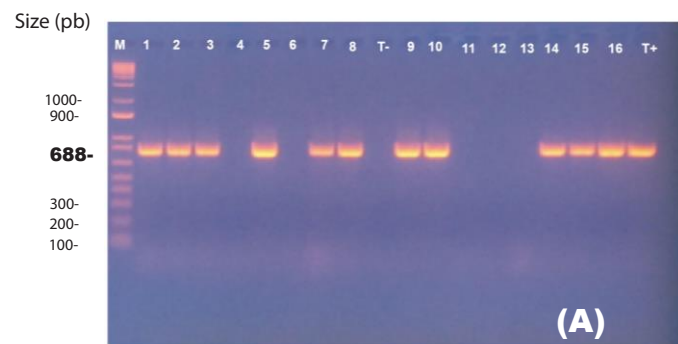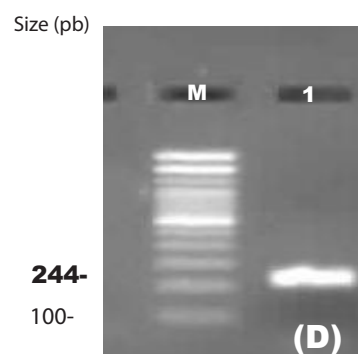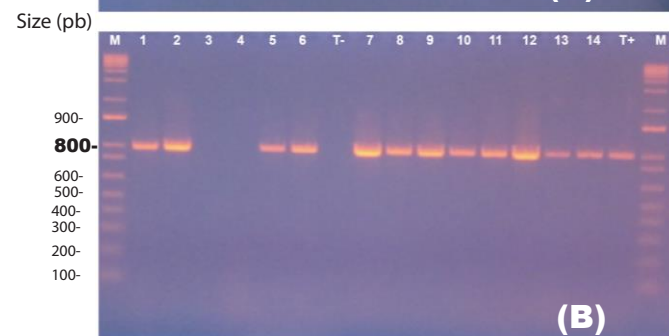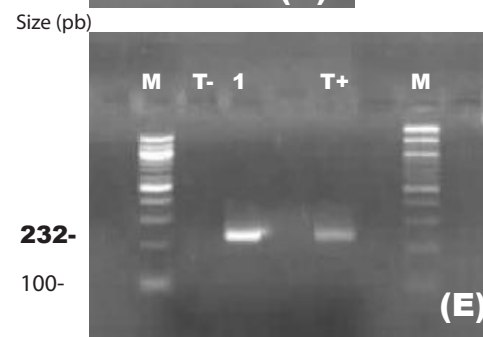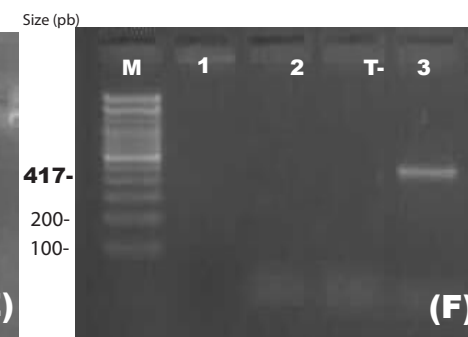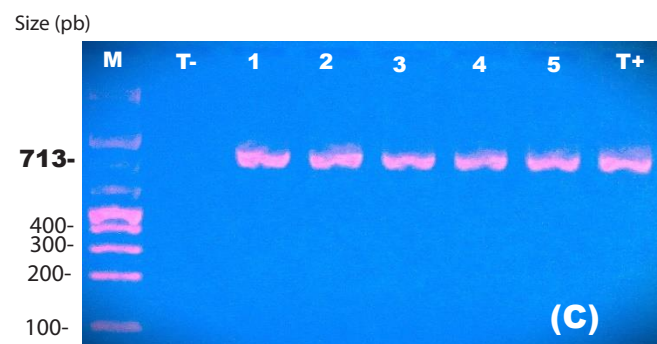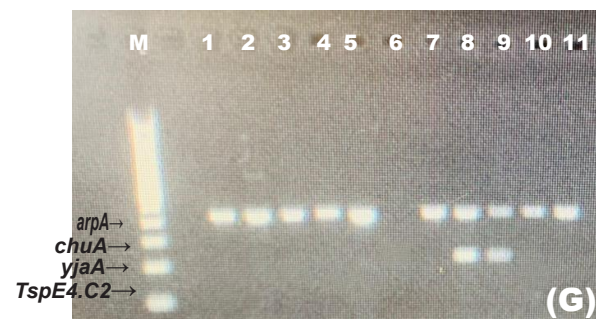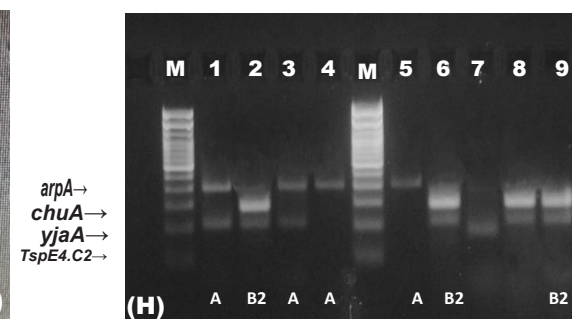

Supplement: S2 File — (PDF) [file pone.0343632.s002.pdf]
